# Small-molecule inhibitor of Gαo for *GNAO1* encephalopathy

**DOI:** 10.1042/BSR20250392

**Published:** 2026-05-13

**Authors:** Yonika A. Larasati, Alexey Koval, Vladimir L. Katanaev

**Affiliations:** 1 Department of Cell Physiology and Metabolism, Faculty of Medicine, University of Geneva, Geneva, Switzerland; 2 Qatar Biomedical Research Institute, College of Health and Life Sciences, Hamad Bin Khalifa University, Doha, Qatar

**Keywords:** drug discovery and design, Gαo, GNAO1, neurodevelopmental disorders, small molecules

## Abstract

*GNAO1*-related neurodevelopmental disorders are caused by mutations in the *GNAO1* gene encoding the major neuronal G protein, Gαo. *GNAO1* encephalopathies manifest in a range of symptoms, including epilepsy, movement disorder, hypotonia, and developmental delay, affecting >400 patients worldwide to date. A growth in the number of diagnosed cases is expected due to the wider availability of whole genome sequencing. One of the most recurrent pathogenic variants causing *GNAO1* encephalopathy is an intronic mutation c.724-8G>A, which results in an in-frame insertion of two amino acid residues, Pro-Gln, after Thr241: Gαo[T241_N242insPQ]. We previously performed in-depth profiling of Gαo[insPQ] using structural, biochemical, and cellular studies. Compared with the wild-type protein, Gαo[insPQ] exhibits faster GTP binding and decreased hydrolysis. Importantly, Gαo[insPQ] is deficient in interacting with regulator of G protein signaling (RGS), GTPase-activating proteins that deactivate Gαo. These defects render Gαo[insPQ] a constitutively active mutant loaded with GTP in the G protein signaling. Patients harboring Gαo[insPQ] variant are in urgent need of novel therapy as they are refractory to available medications. In the present study, we performed a high-throughput screening to find molecules that might suppress the constitutive GTP loading by Gαo[insPQ]. We used a high-diversity chemical library of 54080 compounds, identifying a novel compound, N‐[5‐(2‐methylpropyl)‐1,3,4‐thiadiazol‐2‐yl]‐1H‐1,2,3‐benzotriazole‐5‐carboxamide, that decreases the GTP binding rate of Gαo, likely acting as a competitive inhibitor with higher selectivity to the pathogenic protein. This small-molecule inhibitor of Gαo opens new opportunities to drug discovery towards Gαo-dependent pathologies.

## Introduction

Heterotrimeric G proteins are the main transducers of G protein-coupled receptors (GPCRs), the largest and most versatile receptor class in animals [1]. Composed of Gα and Gβγ, the heterotrimeric complex engages GPCR when Gα is loaded with GDP. Activated GPCRs facilitate GDP to GTP exchange in Gα, promoting dissociation of Gα–GTP from Gβγ, thus acting as guanine nucleotide exchange factors (GEFs). Both Gα–GTP and Gβγ can then engage with distinct downstream effectors, activating second messengers or other downstream pathways. Over time, Gα hydrolyzes its GTP back to GDP, and this GTPase activity can be accelerated by GTPase-activating proteins (GAPs), represented by the regulator of G protein signaling (RGS) family [2]. To complete the loop, GDP-bound Gα then re-associates with Gβγ, forming a heterotrimeric G protein ready to be activated by GPCR in the next cycle. In addition to GEF and GAP activities, a third type of regulatory activity exists for G protein, mediated by guanine nucleotide dissociation inhibitors (GDIs) counteracting nucleotide exchange and G protein activation [3].

Gαo, the most abundant Gα isoform in the brain, is essential for neurodevelopment and neuronal physiology [4]. Despite its central function in the neuronal system, little was known about the exact mechanism of Gαo biological function compared with the other Gα isoforms until recently [5,6]. The broad interest in Gαo came forth after the first diagnosis of *GNAO1* encephalopathies—a broad range of neurological abnormalities caused by mutations in *GNAO1*, the gene encoding Gαo [7,8]. It was first identified as the cause of early onset epileptic encephalopathy in 2013 [7], while further reports show multiple clinical manifestations of *GNAO1* encephalopathies, including epilepsy, movement disorder (MD), developmental delay, and brain atrophy in certain cases [8–10]. More than 400 patients worldwide have been diagnosed with *GNAO1* encephalopathy (https://gnao1.org/), with at least 80 distinct (mostly missense) mutations in *GNAO1* described as “pathogenic” or “likely pathogenic” in the ClinVar database. These numbers are expected to increase with the wider availability of whole exome/genome sequencing.

*GNAO1* encephalopathies represent a highly unmet medical need. Current therapies are symptomatic—for motor dysfunctions they include, e.g., benzodiazepines and levodopa, while for epilepsy they include antiepileptic drugs such as carbamazepine, levetiracetam, or valproic acid [11–13]. Unfortunately, the response to those drugs is sporadic and heterogeneous, with the majority of patients being refractory to those treatments. Deep-brain stimulation (DBS) offers a more effective treatment for patients with movement disorders (but not other symptoms) [14]. However, the high cost and the need for a highly experienced surgical team to perform DBS limit the applicability of this therapy for most patients [15]. Therefore, drug discovery efforts targeting the core of this disease, pathogenic Gαo, are a rational approach of very high interest and need.

About half of the reported cases are in the “hotspot” region of *GNAO1*, affecting codons Gly203, Arg209, and Glu246 [8,12]. We have characterized the biochemical and cellular deficiency of pathogenic variants affecting these three codons: G203R, R209C, and E246K [16]. These mutations accelerate GTP uptake and inactivate GTP hydrolysis, resulting in constitutive GTP binding by Gαo. These mutants also fail to adopt the activated conformation and display aberrant interactions with cellular signaling partners. Among the cellular signaling partners tested are Gβγ and RGS19, a GAP known to accelerate GTP hydrolysis of Gαo [17]. Subsequent studies have shown that varying deficiencies of interactions with these cellular partners, accompanied by increased GTP uptake and reduced GTP hydrolysis rates, are typical features of multiple pathogenic Gαo variants [18–25].

Previously, we successfully launched a high-throughput screening (HTS) to search for small molecules capable of correcting the biochemical deficiency of mutant G203R, R209C, and E246K [16]. From a chemical library of 2736 U.S. Food and Drug Administration (FDA)-approved and pharmacopeial drugs, zinc pyrithione and zinc salts emerged as molecules restoring the GTPase activity of G203R, R209C, and E246K mutants, with negligible effects on wild-type Gαo. In the cellular system, Zn^2+^ restored the ability of the three mutants to interact with various signaling partners. Importantly, we validated the effect of d zinc *in vivo*, where dietary zinc restored the motor function and longevity of the *Drosophila* model of *GNAO1* encephalopathy [16]. These discoveries were followed by safety studies in mouse models and then by the first-in-human clinical application of zinc acetate to a *GNAO1* patient, providing significant improvements in the patient’s conditions [26]. This success is highlighted as one of the important breakthroughs in the development of therapies for pediatric movement disorders [27] and led to a pilot clinical trial currently in completion (NCT06412653).

Another mutation has emerged as a hotspot of *GNAO1* encephalopathy: an intronic mutation c.724-8G>A (NM_020988.3) [11,20,28–33]. This mutation creates a novel splice acceptor site, resulting in an in-frame insertion of two amino acid residues, Pro-Gln, between Thr241 and Asn242: Gαo[T241_N242insPQ] (insPQ mutant). This insertion of the Pro-Gln (PQ) residues is the only known insertion mutation not only for Gαo but also for the entire 16-member family of Gα subunits. Importantly, the Gαo[insPQ] mutant is insensitive to Zn^2+^ treatment [20]. Hence, a molecule capable of restoring the molecular defect of the Gαo[insPQ] mutant is desired as a potential therapeutic agent for patients harboring this pathogenic variant.

### Hypothesis

While our previous attempt to identify a modulator of the pathogenic Gαo[insPQ] variant through HTS of a 2736-compound library of FDA-approved drugs was unsuccessful [20], we hypothesized that screening a larger library of chemically diverse compounds might identify a promising drug candidate. We further hypothesized that such a drug candidate might have unusual mechanism(s) of action. With this hypothesis at the basis, in the current study, we describe an HTS attempting to find a molecule that might restore the molecular defect of the Gαo[insPQ] mutant from a large chemical library of 54080 compounds. This screening resulted in discovery of a compound inhibiting the GTP uptake by Gαo, with higher activity towards the pathogenic variant.

## Methods

### Purification of recombinant Gαo

For production of recombinant Gαo, the Rosetta(DE3)pLysS *Escherichia coli* strain was transformed with pET23b-Gαo wild-type or pET23b-Gαo[T241_N242insPQ] [17,20]. Transformed *E. coli* were then grown at 37°C to OD_600_ = 0.6 before induction with 0.25 mM IPTG and additional growth overnight at 18°C. Cells were then harvested by centrifugation at 3500 × ***g*** at 4°C and resuspended in TBS (20 mM Tris–HCl, pH 7.5, 150 mM NaCl) supplemented with 1 mM PMSF and 30 mM imidazole. Cells were disrupted with high-pressure cell press homogenizer, the debris was removed by centrifugation at 15 000 × ***g***/15 min/4°C. The supernatant was applied to the Ni^2+^ resin (Qiagen) overnight in a rotary shaker at 4°C. The Ni^2+^ resin was washed twice with 10 resin volumes of TBS supplemented with 10 mM imidazole. On the third wash, the washing buffer was supplemented with 3% glycerol, 10 mM MgCl_2_, 0.1 mM DTT, and 200 μM GDP. The Ni^2+^ resin was washed two more times with 10 resin volumes of the washing buffer. Proteins were then eluted with TBS containing 300 mM imidazole.

For production of recombinant GST-RGS4, the Rosetta(DE3)pLysS *E. coli* strain was transformed with pGEX-4T1-RGS4. Transformed *E. coli* was grown and harvested as described above. The supernatant was applied to the Glutathione Sepharose 4B beads (GE Healthcare) overnight in a rotary shaker at 4°C. The beads were washed thrice with 10 resin volumes of TBS. Proteins were then eluted with TBS containing 20 mM glutathione (pH 7.5).

To subsequently remove imidazole or glutathione, the protein buffer was exchanged into TBS using Vivaspin concentrator. Protein concentration was measured using the Bradford assay and the purity was analyzed using SDS–PAGE followed by Coomassie staining.

### BODIPY-GTPγS and BODIPY-GTP assay

The GTP binding and hydrolysis assay using BODIPY-GTP (Invitrogen) or BODIPY-GTPγS (Invitrogen) was performed as described [17,20]. Gαo (1 μM) and RGS4 (1 μM) were diluted in the reaction buffer (TBS supplemented with 10 mM MgCl_2_ and 0.5% BSA). The mixture was then pipetted into black 384-well plates (Greiner) and BODIPY-GTP or BODIPY-GTPγS (1 μM; Invitrogen) was added into the wells. Fluorescence measurements were performed with a Tecan Infinite M200 PRO plate reader with excitation at 485 nm and emission at 530 nm at 28°C. The GTP binding and hydrolysis data of Gαo were fit to obtain the *k_bind_* and *k_hydr_* rate constants as previously described [17,20]. For the BODIPY-GTPγS displacement analysis, 5 μl of compound 1 (to 50 μM) solution in TBS was injected into 20 μl of the BODIPY-GTPγS-loaded Gαo solution at the indicated time without interruption of measurements.

### High-throughput screening

HTS for mutant Gαo modulators was performed using the Gαo[insPQ] and RGS4 proteins. Chemical library was Enamine high-diversity GPCR Library (GPR-54-Y-50, Enamine). DMSO or compounds in DMSO (25 μM) were mixed with Gαo[insPQ] and RGS4 at 1 μM, and baseline fluorescence was measured for 30 s before addition of BODIPY-GTP (1 μM) as described in GTP binding assay above. The remaining reaction was carried out for 15 min. HTS was performed using FDSSμCELL Plate Imager (Hamamatsu) provided by RE.A.D.S Unit, University of Geneva; while hit validation was performed using Tecan Infinite M200 PRO plate reader as described in GTP binding assay above.

Compound 1 used in the hit validations was commercially available from Enamine (Z243139180).

### Europium-GTP assay

Europium-GTP (Eu-GTP) was from the DELFIA Eu-GTP binding kit (PerkinElmer Life Sciences), performed as described [34]. Briefly, components were mixed in appropriate volume in the AcroWell 96 GHP filter plate (Pall) wells. Final concentrations were 50 mM Hepes, 50 mM NaCl, 1 mM MgCl_2_, 10 nM of Gαo, and compound 1 (1 μM or 10 μM). Reaction was started by the addition of Eu-GTP to a final concentration of 10 nM. After an incubation time as indicated in Figure 2G on a horizontal shaker (150 rpm) at room temperature, reaction mixtures were directly filtered on a Millipore Multiscreen HTS Vacuum Manifold and washed thrice with 150 μl of ice-cold washing solution (25 mM Tris–HCl, 100 μM MgCl_2_). Plates were immediately measured in a Tecan Infinite M200 PRO plate reader using the standard protocol for Eu time-resolved fluorescence.

### BRET assay for Gαo-βγ complex formation and dissociation

The bioluminescence resonance energy transfer (BRET) assay was performed as described [16]. Briefly, HEK293T cells were transfected with the Go1-CASE plasmid [35] encoding nanoluciferase-tagged Gαo, Gβ3, and Gγ9 with the Venus tag, along with the plasmid encoding M2 muscarinic receptor (cDNA Resource Center, #MAR0200000). Acetylcholine (10 μM) was injected to activate the receptor. Tecan Infinite M200 PRO plate reader was used to measure the BRET.

### Molecular docking

Molecular docking simulations were performed using AutoDock Vina to predict the binding orientation of Compound 1 within the Gαo active site. The Gαo structural model was used as previously described [21]. Compound 1 was docked using standard force field parameters and a grid box of 60 Å × 65 Å × 61 Å centered on the canonical nucleotide-binding pocket. To account for induced-fit effects and the active site’s plasticity, flexible residue docking was employed for residues within 5 Å of the binding interface, encompassing the entire GTP-binding domain. Following the simulation, the binding pose corresponding to the most negative binding affinity score in kcal/mol was selected for detailed interaction analysis and figure preparation.

## Results and discussion

### High-throughput screening

**Figure 1:**
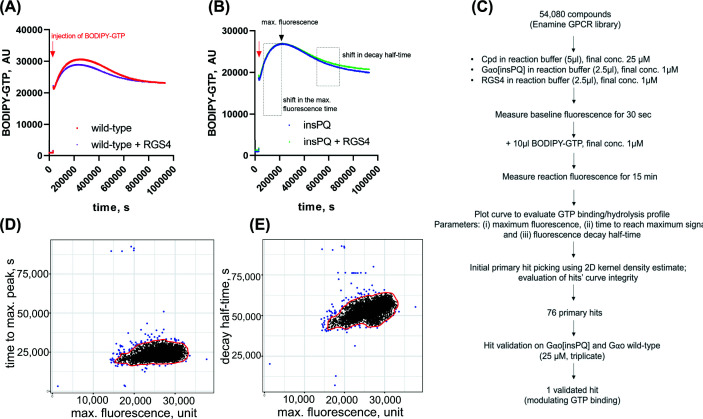
High-throughput screening (HTS) for the insPQ mutant. High throughput screening (HTS) for the insPQ mutant. **(A,B)** Representation of BODIPY-GTP curves from control wells in HTS. (**A**) Gαo wild-type (1 μM) in the absence or presence of RGS4 (1 μM), (**B**) Gαo insPQ (1 μM) in the absence or presence of RGS4 (1 μM). Three parameters (max. fluorescence, time to achieve max. fluorescence, and decay half-time) analyzed from the screening are shown in panel (B). (**C**) Workflow of HTS performed in the present study. Cpd—compound. (**D,E**) HTS results plotted by the three screening parameters. For hit picking, zones containing 95% of points were identified using 2D kernel density estimate (red contour), and the points outside of it (in blue) were considered as initial primary hits. See the main text for more details.

To evaluate the GTP binding/hydrolysis properties of Gαo, we employed *in vitro* biochemical assays using recombinant Gαo and BODIPY-labeled guanine nucleotides: (i) BODIPY-GTPγS—a non-hydrolyzable GTP analog—to measure GTP binding rate and (ii) BODIPY-GTP to measure GTP hydrolysis [16,17,20,36]. We have previously extensively characterized the biochemical properties of the Gαo[insPQ] mutant, in which insertion of amino acids PQ occurs at the beginning of switch III of Gαo and results in misconfigured switch III of Gαo [20]. Consequently, Gαo[insPQ] exhibits an increased GTP binding and decreased GTP hydrolysis rates [20]. Moreover, the Gαo[insPQ] mutant is less sensitive to the effect of RGS protein on GTP hydrolysis *in vitro* (Figure 1A,B) and fails to bind RGS protein in the cellular setting [20]. These properties suggest the Gαo[insPQ] mutant might have a prolonged GTP-bound state compared with wild-type Gαo.

**Figure 2:**
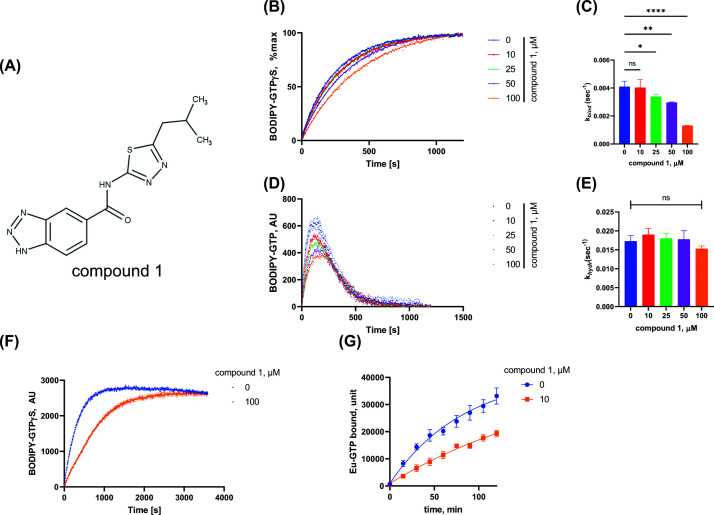
Compound 1 inhibits GTP binding by wild-type Gαo. Compound 1 inhibits GTP binding by Gαo**.** (**A**) Structure of compound 1. (**B,C**) Gαo (1 μM) was treated with different concentrations of compound 1 before being subjected to the BODIPY-GTPγS assay. (B) BODIPY-GTPγS curves, (C) Calculation of apparent BODIPY-GTPγS binding rates (*k_bind_*) of curves in panel (B). (**D,E**) Gαo (1 μM) was treated with different concentrations of compound 1 before being subjected to the BODIPY-GTP assay. (D) BODIPY-GTP curves, (E) Calculation of apparent BODIPY-GTP hydrolysis rates (*k_hydr_*) of curves in panel (D). (**F**) Gαo (1 μM) was treated with vehicle or 100 μM of compound 1 before being subjected to the BODIPY-GTPγS assay for longer time (1 h). (**G**) Gαo (10 nM) was treated with vehicle or 10 μM of compound 1 before addition of Eu-GTP (10 nM) at the indicated time points. Fluorescence of bound Eu-GTP was measured as described in Methods. Data in panels (B–G) are average ± SEM (*n* ≥ 3). Statistical analysis in panels (C) and (E) was performed using one-way ANOVA followed by Dunnet’s multiple comparison test. ns: not significant, ∗*P* <0.05, ∗∗*P* <0.01, and ∗∗∗∗*P* <0.0001.

We have previously optimized a HTS pipeline to screen an FDA-approved drug library of 2736 compounds for drugs that could restore Gαo[insPQ] mutant’s sensitivity toward RGS4, which has not resulted in an adequate repurposing drug candidate [20]. Using the same pipeline, in the present study, we launched an HTS campaign using 54080 compounds from a high-diversity chemical library (Figure 1C). As BODIPY-GTP assay is a multiparametric assay, assay performance cannot be fully described by a single parameter such as Z’-factor. We then analyzed three parameters from the data obtained in the primary screening, looking for compounds that would either (i) change the fluorescence maximum in the BODIPY-GTP assay, or (ii) shift the peak of maximal fluorescence in time, or (iii) affect the half-time in the fluorescence decay phase of the assay (Figure 1B,D,E). Initial primary hits were cherrypicked based on either of these parameters. The integrity of the hits’ curves was examined to ensure the absence of any indications of artifacts, such as bubbles in the wells, local plate aberrations, or hits affecting assay fluorescence. Seventy-six primary hits were identified and retested in triplicate at 25 μM, using both Gαo wild-type and Gαo[insPQ] in the presence of RGS4.

Among these primary hits, only one was confirmed to be a “true hit” (compound 1, N‐[5‐(2‐methylpropyl)‐1,3,4‐thiadiazol‐2‐yl]‐1H‐1,2,3‐benzotriazole‐5‐carboxamide, Figure 2A), affecting the BODIPY-GTP and BODIPY-GTPγS binding of both Gαo[insPQ] and Gαo wild-type in the presence of RGS4 (Supplementary Figure S1). Compound 1 also affected the binding of BODIPY-GTP and BODIPY-GTPγS binding to Gαo wild-type and Gαo[insPQ] in the absence of RGS4 (Supplementary Figure S2). This indicates that the compound modulates the general GTP binding/hydrolysis of both Gαo wild-type and Gαo[insPQ], but the effects towards Gαo[insPQ] were more profound (Supplementary Figures S1, S2).

Other primary hit compounds were either false positives or compounds that affect BODIPY fluorescence. The low number of validated hits likely reflects the use of a high-diversity compound library, which reduces redundancy in chemical scaffolds and typically results in lower hit rates. In addition, the screening was performed using purified Gαo protein, providing a highly specific biochemical readout and minimizing indirect or off-target effects that are more commonly observed in cell-based assays.

### Compound 1 inhibits the GTP uptake rate of Gαo

To further validate the activity of compound 1, we ordered a new batch of the compound and re-tested it against Gαo wild-type. Compound 1 inhibited the BODIPY-GTPγS binding rate in a dose-dependent manner (Figure 2B,C), while the hydrolysis rate of BODIPY-GTP remained unaffected (Figure 2D,E). Longer duration of the assay highlighted the reduced rate of BODIPY-GTPγS uptake in the presence of compound 1 even more vividly (Figure 2F). Compound 1 also dose-dependently inhibited BODIPY-GTPγS binding rate to pathogenic Gαo[insPQ] (Supplementary Figure S3A,B). However, as the apparent *k_bind_* of wild-type Gαo did not reach a plateau upon compound treatment, even at concentrations up to 100 μM, we were unable to reliably determine and compare IC_50_ values across the recombinant proteins. To better visualize the effect of compound 1, we normalized the apparent *k_bind_* of each protein in the presence of compound to its respective untreated control (Supplementary Figure S3C). This analysis demonstrates that the Gαo[insPQ] mutant exhibits greater sensitivity to compound 1. Furthermore, we also observed that compound 1 inhibits BODIPY-GTPγS binding rate to recombinant Gαi1 (Supplementary Figure S4A, B). The calculated apparent *k_bind_* and *k_hydr_* values are shown in Supplementary Table S1.

To exclude the possibility that the effect of compound 1 was restricted to the bulky BODIPY-GTPγS or was due to an unspecific effect on the fluorescence property of BODIPY moiety, we evaluated GTP uptake using another chemical probe, Eu-GTP, a poorly hydrolyzable GTP analog labeled by Europium chelate [34]. In the Eu-GTP binding assay, compound 1 decreased Eu-GTP incorporation rate towards Gαo (Figure 2G)—an effect that was similar to that induced by well-characterized GDIs such as Gβγ or GoLoco peptide [34]. As Eu-GTP and BODIPY-GTPγS carry their labels at distinct positions on the nucleotide scaffold and have different photophysical properties, the effect of compound 1 observed in both assays was unlikely to result from artifact of fluorescent probe.

### Compound 1 likely acts as a competitive GTP inhibitor

The capacity of compound 1 to reduce the rate of GTP uptake by Gαo raised the question of its precise mechanism of action. One possibility is that it acts as a GDI, analogously to what has been proposed for certain Gαq modulators [37]. GDI-like compounds—of which YM-254890 and FR900359 are the best-characterized examples—bind allosterically to the GDP-bound form of Gα, stabilizing the inactive conformation and preventing nucleotide exchange without directly occupying the guanine nucleotide–binding pocket [37,38]. Alternatively, compound 1 might act as a direct competitive inhibitor, engaging the nucleotide-binding site and displacing GTP through steric or energetic competition.

Evaluation of the binding mode of compound 1 within the catalytic active site using molecular docking revealed that compound 1 fits well into the nucleotide-binding pocket. The overall configuration is reminiscent of the native ligand, GTP (Supplementary Figure S5). Interestingly, the orientation of the scaffold within the pocket was unexpected. The benzotriazole moiety, which has a stronger structural similarity to the guanine base, unexpectedly occupies the region usually reserved for the α, β, and γ-phosphate groups of GTP. In contrast, it is the thiadiazole group that aligns with the guanine-binding moiety. Despite the overall spatial similarity of the binding pose, compound 1 does not fully replicate the canonical hydrogen-bonding network utilized by GTP. The “phosphate equivalent” benzotriazole of the compound forms a critical hydrogen bond with S47 of the P loop but not with the equally important T48 residue [39]. The thiadiazole and the adjacent carboxamide form strong interactions with R177, R179, and S152; however, none of these residues are involved in GTP binding, which is mostly held by D273 and K271 [40]. The isobutyl substituent of the thiadiazole ring, however, preserves a hydrophobic link with L274. Thus, these data demonstrate that, while compound 1 mimics the volume and general orientation of GTP, it achieves binding through a non-canonical interaction fingerprint.

**Figure 3:**
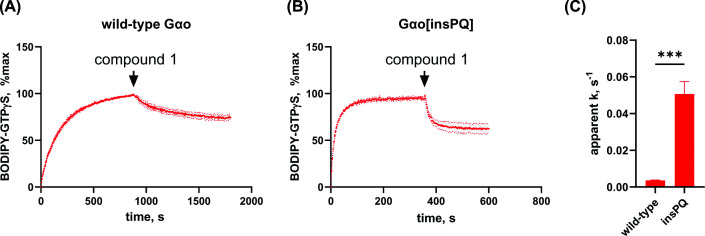
Compound 1 acts as a putative competitive GTP inhibitor of Gαo subfamily, with more pronounced effects on pathogenic Gαo. Compound 1 acts as a putative competitive GTP inhibitor of Gαo subfamily, with more pronounced effects on pathogenic Gαo. (**A,B**) Representative graphs of wild-type Gαo (A) and Gαo[insPQ] (B) loading by BODIPY-GTPγS followed by injection of compound 1 (indicated by arrowhead), which results in displacement of the fluorescence ligand and drop in fluorescence. (**C**) Quantification of apparent rate constants of BODIPY-GTPγS displacement upon outcompeting by compound 1. The data are presented as an average from *N* = 3–6 independent experiments, SEM is indicated as filled area around the curve or error bars. Statistical assessment performed by t-test, ***P <0.001.

To distinguish experimentally between the two possible mechanisms, we exploited a key functional difference: because GDI-like compounds do not occupy the nucleotide-binding pocket, they cannot displace pre-bound fluorescent GTP analogues in displacement assays, whereas a competitive inhibitor acting at the same site would be expected to do so. We therefore tested the ability of compound 1 to displace BODIPY-GTPγS pre-loaded onto wild-type and Gαo[insPQ] by injecting it during an ongoing nucleotide-loading experiment. This approach has been previously applied to identify inhibitors targeting nucleotide-binding sites in other G proteins, such as small GTPases Rab7 and Rac1 [41,42]. As shown in Figure 3A,B, addition of compound 1 produced a significant drop in fluorescence, demonstrating displacement of the pre-bound BODIPY-GTPγS analogue. This result is inconsistent with a GDI-like mechanism and instead supports direct competition at the guanine nucleotide–binding site.

Notably, the rate and extent of displacement were significantly more pronounced for the pathogenic Gαo[insPQ] variant compared with wild-type Gαo (Figure 3C), suggesting differential susceptibility of the mutant protein to competitive inhibition—a finding with potential implications for selective targeting of the pathogenic variant. Compound 1 also displaced BODIPY-GTPγS from wild-type Gαi_1_ with a rate and extent comparable to those observed for wild-type Gαo (Supplementary Figure S4C), consistent with the high sequence conservation of the nucleotide-binding pocket across Gαi/o family members and indicating that selectivity within this subfamily will require further optimization.

To our knowledge, compound 1 is the first small molecule found to act as a Gαo inhibitor. Small molecule inhibitors have been previously discovered for Gαq. YM-254890, a macrocyclic desipeptide isolated from *Chromobacterium sp*. QS3666, blocks GDP release from the Gαq protein and hence inhibits GDP/GTP exchange in Gαq [37,38]. The GDI effect of YM-254890 manifests in the strong inhibition of intracellular Ca^2+^ mobilization and serum response element-mediated transcription stimulated by several receptors coupled to Gαq, but not those coupled to Gαi, Gαs, or Gα15 [37]. Another macrocyclic peptide isolated from the plant *Ardisia crenata*, FR900359, is a natural structural analog of YM-254890 [43]. FR900359 also selectively inhibits Gαq/11-induced signaling, which translates to its efficacy in attenuating Gαq-driven oncogenic signaling [44]. Subsequent studies questioned both the selectivity of these compounds towards Gαq/11, suggesting that YM-254890 also inhibits Gαs and Gαi [45], and the GDI mode of action, showing that YM-254890 and FR900359 prevent dissociation of Gα and Gβγ [46].

To test whether compound 1 might similarly affect Gαo-βγ interactions, we employed a BRET assay measuring basal levels of the heterotrimeric complexes, and M2 muscarinic receptor-induced dissociation of Go, in control and compound 1-treated cells. In these experiments, we found that different concentrations of compound 1 failed to affect Gαo-βγ interactions, both in the basal and stimulated conditions (Supplementary Figure S6). However, we cannot exclude that compound 1 shows limited cellular activity under the conditions tested, potentially due to insufficient membrane permeability or intracellular availability.

Importantly, the preferential activity of compound 1 toward the pathogenic Gαo variant highlights its value as a biochemically validated chemical probe and starting scaffold for further optimization. Future studies will be required to improve its cellular properties and to evaluate its effects in more complex cellular and disease-relevant models.

### Study limitations

Although biochemical assays using two fluorescent nucleotide probes are consistent with compound 1 acting as a putative competitive inhibitor of GTP binding, biophysical and structural studies—such as X-ray crystallography, cryo-EM, or surface plasmon resonance—will be required to conclusively define its binding mode and confirm direct engagement at the guanine nucleotide–binding pocket.

The guanine nucleotide–binding pocket is highly conserved across Gα subunits; therefore, potential activity toward other Gα families (e.g., Gαs and Gαq/11) cannot be excluded. In addition, inhibition of Gαi/o proteins may lead to indirect, system-level effects on downstream signaling pathways (e.g., modulation of cAMP levels), which were not assessed in the present study.

Another limitation is the lack of direct assessment of compound 1 cellular uptake and intracellular availability. This currently precludes robust evaluation in downstream functional assays, such as second messenger readouts or neuronal activity measurements.

Finally, *GNAO1* encephalopathy is an ultra-rare disorder, with approximately 400 diagnosed patients worldwide. Although the intronic *GNAO1* mutation c.724-8G>A, leading to expression of the Gαo[insPQ] variant, represents a recurrent hotspot mutation, the overall patient population remains very limited to ensure sufficient investment into the conventional drug development of compound 1 as a potential targeted treatment.

### Future directions

Most pathogenic variants of Gαo responsible for *GNAO1* encephalopathy exhibit increased GTP uptake rates and decreased GTP hydrolysis rates, indicating a tendency toward a constitutively GTP-loaded state of these mutants [16,18,19]. As compound 1 decreases the GTP binding rate of the Gαo[insPQ] mutant, it remains to be determined whether it also acts on other pathogenic Gαo variants. Notably, our data further show that compound 1 inhibits GTP binding to Gαi_1_ to an extent comparable to that observed for wild-type Gαo, suggesting that its activity may extend beyond Gαo to other members of the Gαi/o subfamily. Gα-pathies encompass a broad spectrum of disorders affecting different organs and tissues [47]; therefore, it will be important to determine the selectivity profile of compound 1 across pathogenic variants within the Gαi/o family as well as other Gα subfamilies. Future biophysical, structural, physicochemical, and cellular studies will be required to define the binding mode, assess cellular target engagement and permeability, and evaluate functional consequences on downstream signaling pathways. Altogether, these studies will be essential to clarify the mechanism of compound 1 and to properly assess its selectivity and translational potential as a starting chemical scaffold targeting Gαi/o proteins.

## Supplementary Material

Supplementary Figures S1-S6 and Table S1

## Data Availability

All data generated in this work are available in the manuscript and its supplementary figures.

## Open Access

Open access for this article was enabled by the participation of the University of Geneva in an all-inclusive Read & Publish agreement with Portland Press and the Biochemical Society.

## Abbreviations

BRET: Bioluminescence resonance energy transfer
DBS: Deep-brain stimulation
Eu-GTP: Europium-GTP
FDA: Food and Drug Administration
GAPs: GTPase-activating proteins
GDIs: Guanine nucleotide dissociation inhibitors
GEFs: Guanine nucleotide exchange factors
GPCRs: G protein-coupled receptors
HTS: High-throughput screening
MD: Movement disorder
RGS: Regulators of G protein signaling
