# Supplementary material for: Small-molecule inhibitor of Gαo for GNAO1 encephalopathy
Source: Biosci Rep. 2026 May 13;46(5):BSR20250392. doi: 10.1042/BSR20250392 (PMC13185127; doi:10.1042/BSR20250392)
Supplement: Supplementary Figures S1-S6 and Table S1 [file BSR-2025-0392_supp.pdf]

Supplementary materials (Figures S1-S6, Table S1).

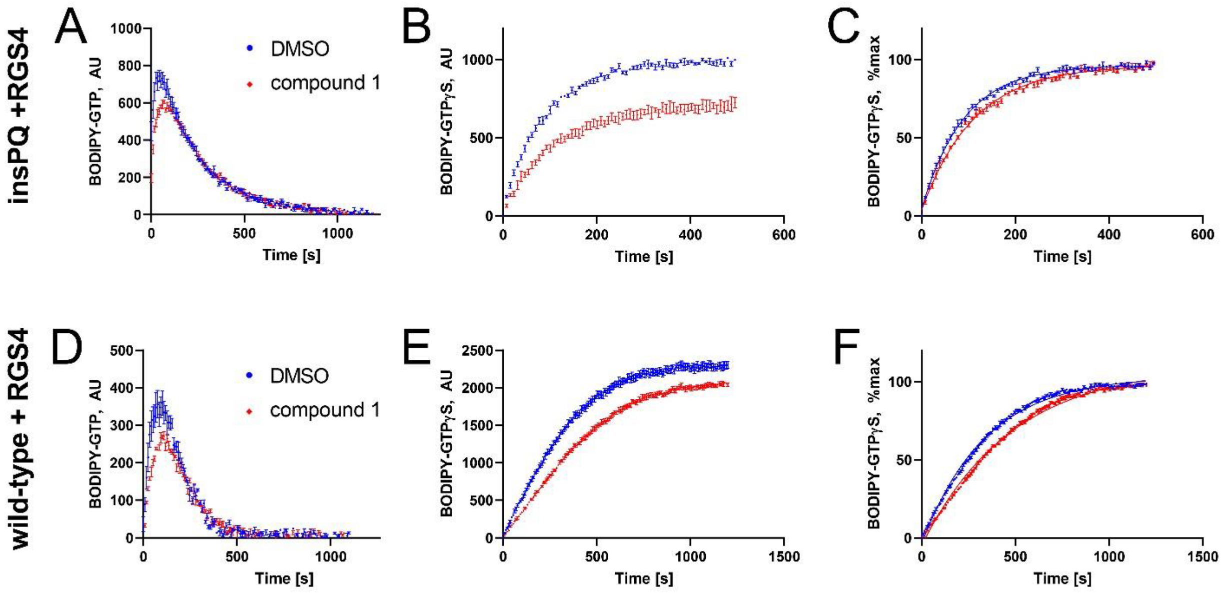

Fig.S1. Effects of compound 1 on Gαo variants in the presence of RGS4. Pathogenic Gαo[insPQ] (A-C) or Gαo wild-type (D-F) were assessed in the BODIPY-GTP assay (A, D) and BODIPY-GTPγS assay (B, C, E, F) in the presence of 1 μM RGS4, upon addition of DMSO or compound 1 (25 μM). Raw fluorescence values are shown as AU (arbitrary units, A, B, D, E) or upon normalization to % of the maximal reached levels (C, F). Data are shown as average ± SEM (n ≥ 3).

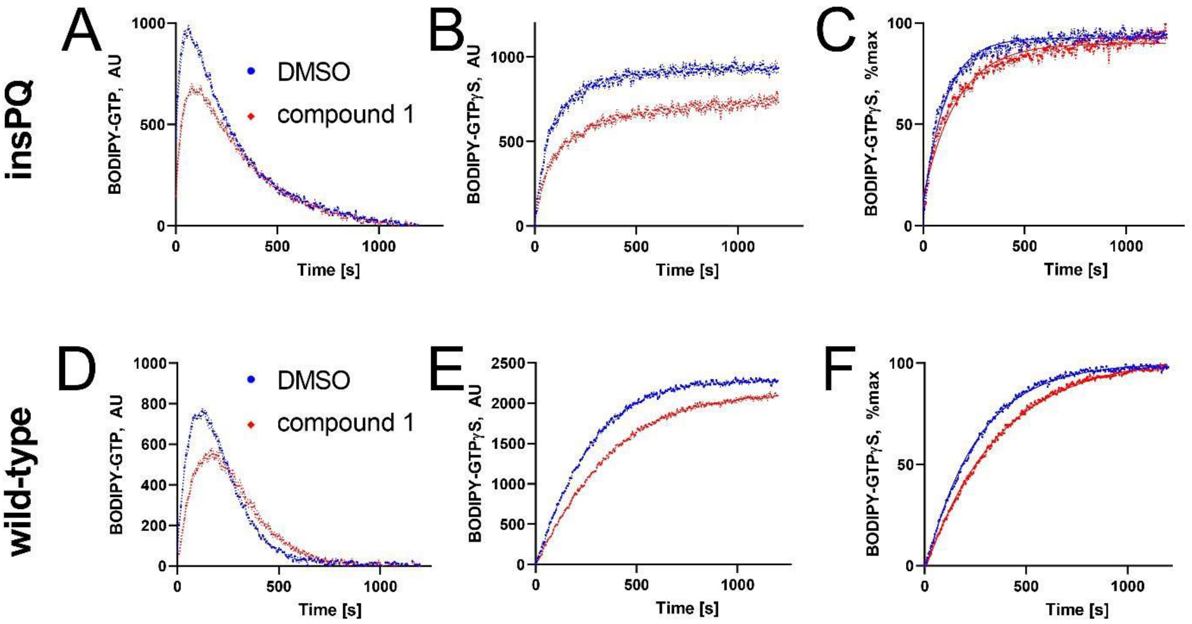

Fig. S2. Effects of compound 1 on Gαo variants in absence of RGS4. Pathogenic Gαo[insPQ] (A-C) or Gαo wild-type (D-F) were assessed in the BODIPY-GTP assay (A, D) and BODIPY-GTPγS assay (B, C, E, F), upon addition of DMSO or compound 1 (25 µM). Raw fluorescence values are shown as AU (arbitrary units, A, B, D, E) or upon normalization to % of the maximal reached levels (C, F). Data are shown as average ± SEM (n ≥ 3).

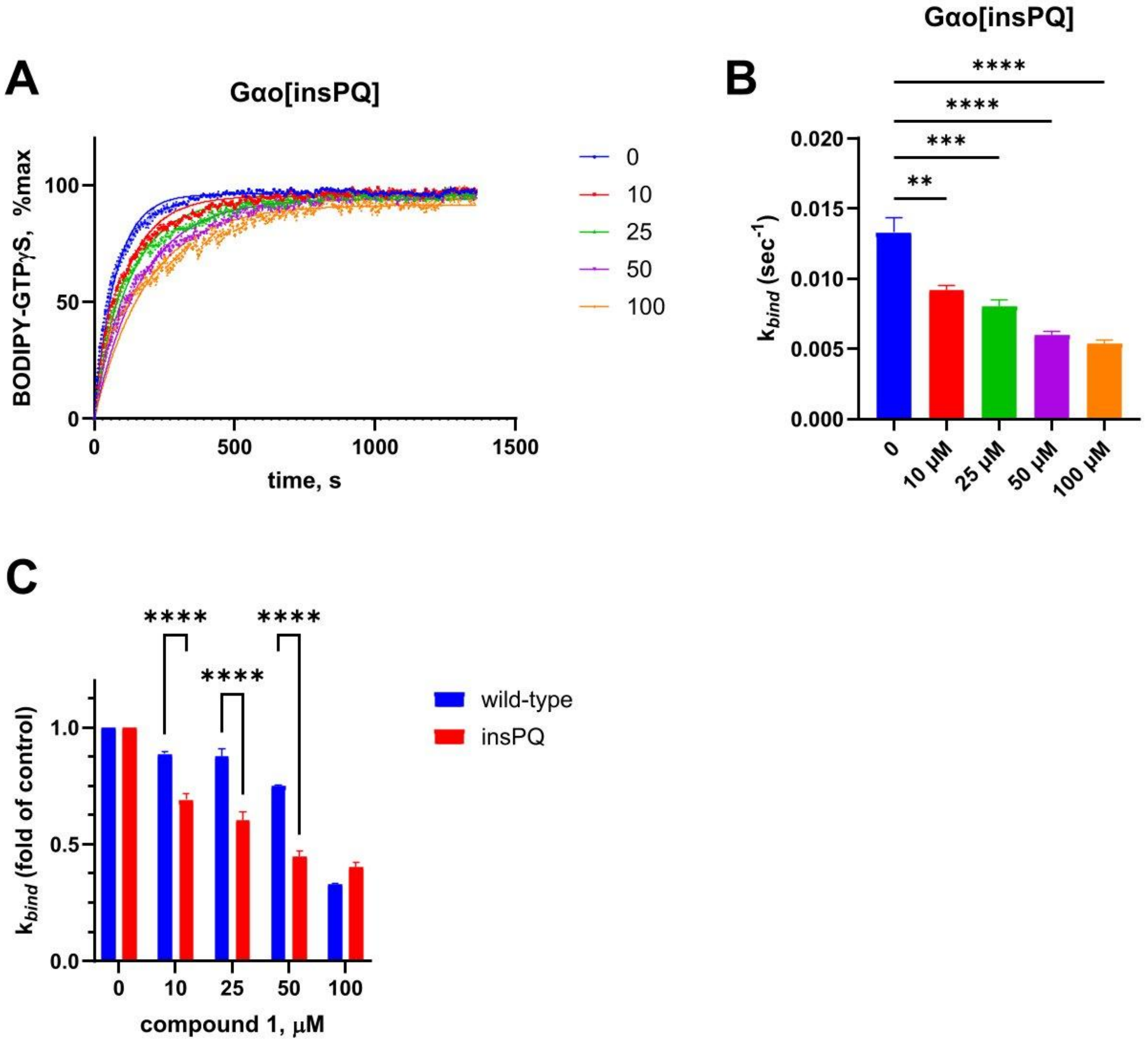

Fig. S3. Compound 1 inhibits GTP binding by pathogenic Gαo[insPQ]. (A and B) Gαo (1 μM) was treated with different concentrations of compound 1 before subjected to the BODIPY-GTPγS assay. (A) BODIPY-GTPγS curves, (B) Calculation of apparent BODIPY-GTPγS binding rates (k_{bind}) of curves in panel A. (C) To compare the effect of compound 1 on wild-type vs pathogenic Gαo, normalization of the apparent k_{bind} of each protein in the presence of compound to its untreated control was performed. Data are average ± SEM (n ≥ 3). Statistical analysis in panel B was performed using one-way ANOVA followed by Dunnet's multiple comparison test. Statistical analysis in panel C was performed using two-ways ANOVA followed by Sidak's multiple comparison test. ns: not significant, **$p < 0.01$, ***$p < 0.001$, and ****$p < 0.0001$.

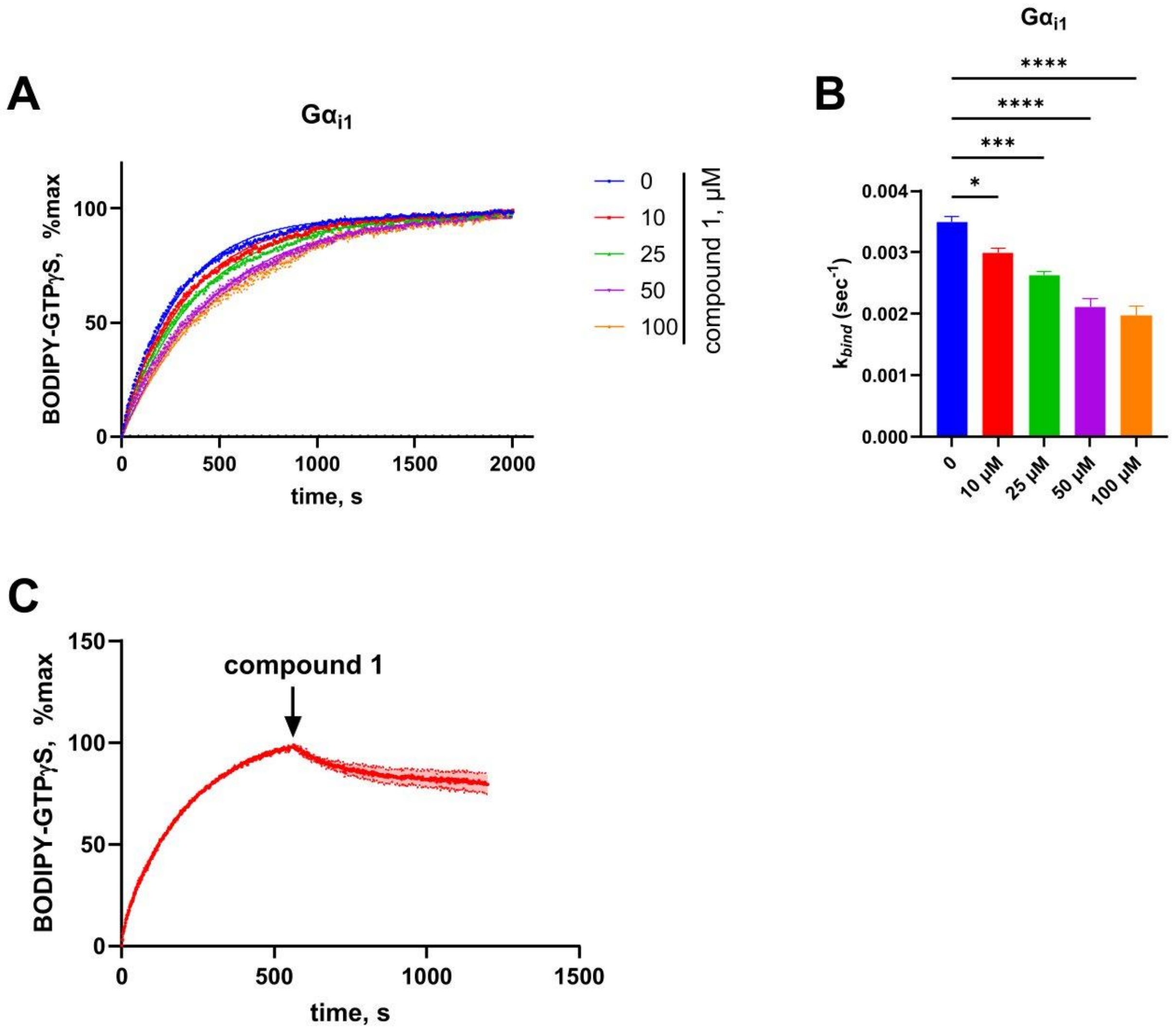

Fig. S4. Compound 1 inhibits GTP binding by wild-type Gαi1. (A and B) Gαi1 (1 μM) was treated with different concentrations of compound 1 before subjected to the BODIPY-GTPγS assay. (A) BODIPY-GTPγS curves, (B) Calculation of apparent BODIPY-GTPγS binding rates (k_{bind}) of curves in panel A. (C) Representative graphs of wild-type Gαi1 loading by BODIPY-GTPγS followed by injection of compound 1 (indicated by arrowhead), which results in displacement of the fluorescence ligand and drop in fluorescence. Data are average ± SEM (n = 3). Statistical analysis in panel B was performed using one-way ANOVA followed by Dunnet's multiple comparison test. ns: not significant, $*p < 0.05$, $***p < 0.001$, and $****p < 0.0001$.

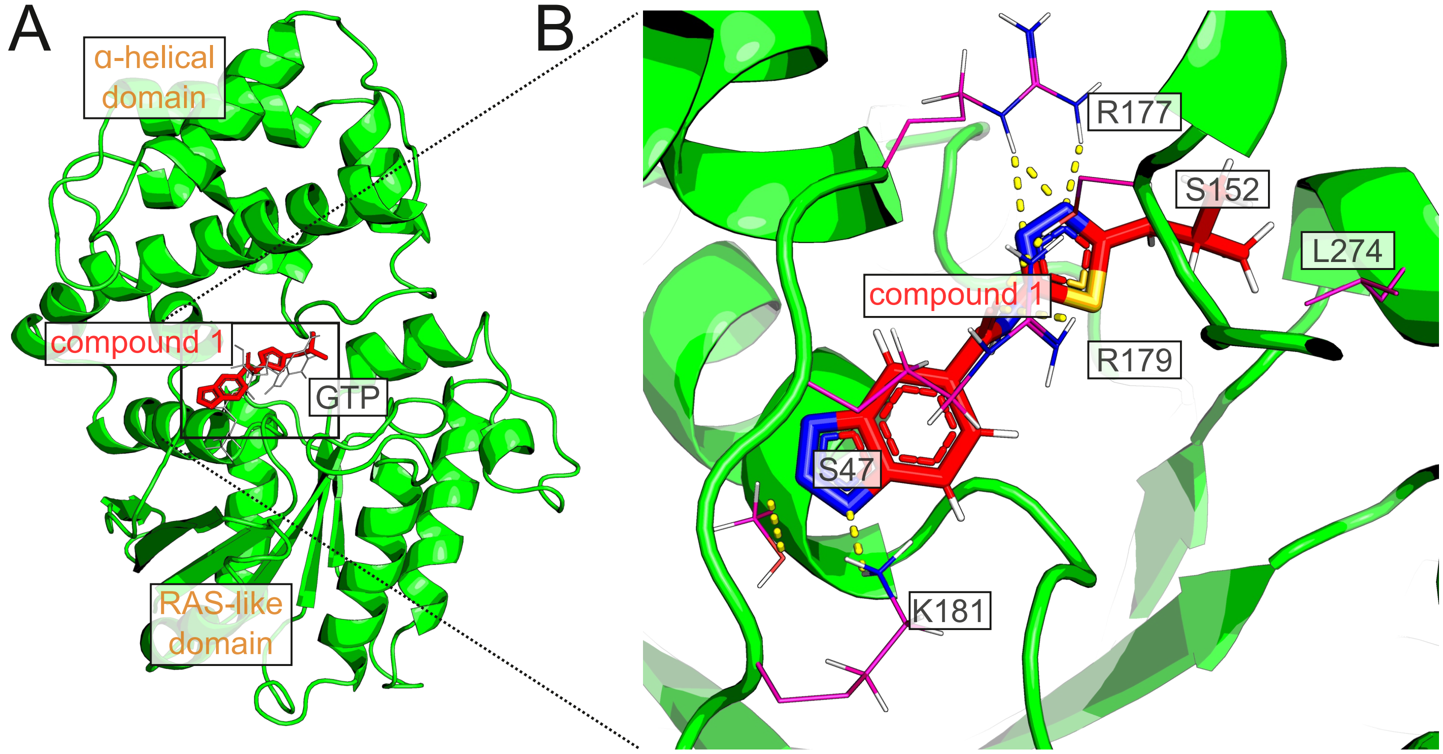

Fig. S5. Binding mode and molecular interactions of compound 1 within the Gαo active site. (A) Overall structural comparison between compound 1 and the native ligand, GTP, in the Gαo catalytic pocket. Compound 1 adopts a configuration overall reminiscent of GTP, with its benzotriazole moiety roughly corresponding to the position of the phosphate groups and the thiadiazole group aligning with the guanine moiety. (B) Detailed view of the binding interface. Compound 1 is stabilized by an extensive network of hydrogen bonds with residues S47, S152, R177, R179, and K181. Additionally, the ligand forms distinct polar contacts with L274. Hydrogen bonds are represented by dashed lines; residues involved in binding are shown as purple sticks.

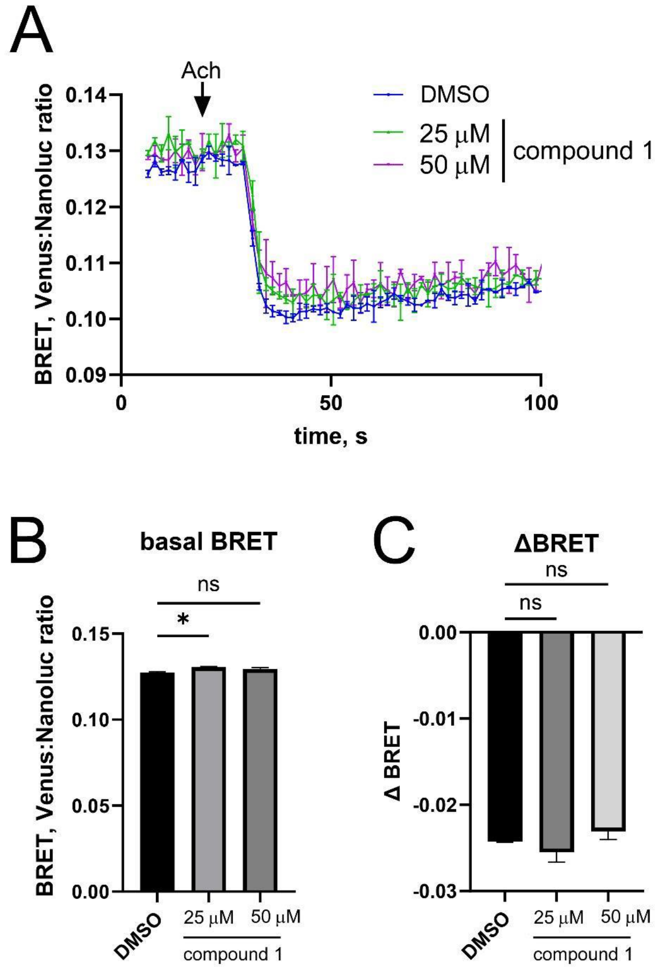

Fig. S6. Compound 1 fails to influence cellular Gαo-βγ interactions. HEK293T expressing GFP-tagged Gαo, Gβ3, Venus-tagged Gγ9, and M2 muscarinic receptor were pretreated with 25 or 50 µM compound 1 for 4 hours prior to the experiment. BRET between GFP-tagged Gαo and Venus-tagged Gγ9 measured the basal level of Gαo-βγ association, decreased upon addition of 10 µM acetylcholine (Ach) (A). Quantification of the basal BRET signal (B), and of its drop upon Ach addition (C) revealed no significant effects of compound 1. Data are from N=2 independent experiments, presented as mean ± SD. Statistical analysis by one-way ANOVA followed by Dunnet's multiple comparison test. ns: not significant, $*p < 0.05$.

Supplementary Table S1. Apparent BODIPY-GTPγS binding rate constants and BODIPY-GTP hydrolysis rate constants of recombinant Gα proteins treated with compound 1

Compound 1	Wild-type Gαo		Gαo[insPQ]	$G\alpha i_1$
	k_{bind} (sec^{-1})	K_{hydr} (sec^{-1})	k_{bind} (sec^{-1})	k_{bind} (sec^{-1})
0	0.004098±0.0001583	0.01726±0.0008527	0.01331±0.001062	0.003496±9.493e-005
10 μM	0.004025±0.0002319	0.01901±0.0009413	0.009195±0.0003464	0.002990±7.921e-005
25 μM	0.003389±6.703e-005	0.01901±0.0007842	0.008025±0.0005043	0.002634±6.217e-005
50 μM	0.002969±2.114e-005	0.01776±0.001329	0.005976±0.0002840	0.002118±0.0001226
100 μM	0.001309±1.299e-005	0.01529±0.0004246	0.005353±0.0002582	0.001969±0.0001536
